# Hibernation in Reeves’ Turtles (*Mauremys reevesii*) in Qichun County, Hubei Province, China: Hibernation Beginning and End and Habitat Selection

**DOI:** 10.3390/ani12182411

**Published:** 2022-09-14

**Authors:** Rongping Bu, Zihao Ye, Haitao Shi

**Affiliations:** Ministry of Education Key Laboratory for Ecology of Tropical Islands, College of Life Sciences, Hainan Normal University, Haikou 571158, China

**Keywords:** Reeve’s turtle, hibernation, habitat selection, conservation, overwintering

## Abstract

**Simple Summary:**

This study investigated Reeves’ turtle (*Mauremys reevesii*) using radiotelemetry to determine the beginning and end dates of, and habitats selected for, hibernation, and to inform future conservation strategies for protection of this species during the hibernation season. Hibernation began in late October 2021 and arousal began in March 2022. Reeves’ turtles mainly hibernate in abandoned ponds or lands. The terrestrial hibernation sites had more herbage cover and were close to the field edge, and the aquatic hibernation sites were covered with herbage, which provides shelter and protection and thermal stability for the turtles during hibernation. We suggest vigilantly protecting this unique resource to provide Reeves’ turtles with secure hibernaculum sites and avoiding redevelopment of these areas during hibernation.

**Abstract:**

Hibernation protects turtles from extreme winter conditions. Reeves’ turtle (*Mauremys reevesii*) is a medium-sized aquatic turtle that lives in freshwater habitats in lowland areas with still or slowly moving water. Currently, little is known regarding its overwintering behavior. In the current study, 20 Reeves’ turtles from the wild were investigated using radiotelemetry in the field to determine the beginning and end dates of, and habitat selected for, hibernation. Hibernation began in late October 2021 and arousal began in March 2022. Reeves’ turtles do not appear to be limited in their selection of suitable hibernation habitats, which included fish ponds, abandoned ponds (ponds not being used for farming), marshes, and abandoned fields (fields not being used for farming). In the aquatic hibernation habitats, only herbage cover was significantly different between the selected and random habitats (*t* = 2.525, *df* = 9, *p* = 0.033). In the terrestrial hibernation habitats, there were significant differences in the canopy (*Z* = −2.201, *p* = 0.028), slope gradient (*Z* = −2.032, *p* = 0.042), herbage cover (*Z* = −2.379, *p* = 0.017), and distance from the habitat edge (*Z* = −2.524, *p* = 0.012) between the selected and random habitats. This indicates that Reeves’ turtles prefer to hibernate at the soft edges of flat habitats with low canopy and high herbage cover when hibernating in terrestrial habitats and prefer to hibernate at sites with high herbage cover when hibernating in aquatic habitats. To the best of our knowledge, this is the first study to investigate hibernation in wild Reeves’ turtles in the field, and the results identify key ecological variables correlated with habitat selection during hibernation. This knowledge could inform local conservation measures related to farming activities.

## 1. Introduction

The physiological processes and behaviors of turtles are closely related to their environment [1,2], and hibernation is a crucial aspect of their life history [3]. As turtles are ectotherms, their metabolism is considerably reduced at low temperatures [4]. Hibernating turtles have low activity levels, reduced metabolic rates, and no food intake [5,6], and face three main risks during hibernation: hypoxia, freezing, and predation [4,7,8].

Turtles use different strategies to cope with adverse conditions during hibernation. Species that overwinter in anoxic conditions may use eutrophic wetlands, whereas those that are relatively intolerant of anoxic conditions must be able to access well-oxygenated microenvironments, such as those that occur in lakes and rivers, to survive in winter [4,9]. However, aquatic habitats may dry up in winter or have significantly reduced water levels, which may pose a greater predation risk to turtles [10]. Many turtle species that hibernate terrestrially face physical challenges associated with leaving the water, such as the risk of freezing or dehydration [10]. However, as long as they are able to overcome freezing or dehydration—by excavating or occupying burrows or burying themselves in mud—hibernation on land poses fewer risks of hypoxia and predation [4,10,11].

Reeves’ turtle (*Mauremys reevesii*) (Geoemydidae) is a medium-sized turtle species that is widely distributed in East Asia throughout central and eastern continental China, southern Japan, and part of the Korean peninsula [12]. This species lives in freshwater habitats in lowland areas with still or slowly moving water. The population of Reeves’ turtles has experienced a significant decline over the last couple of decades due to habitat destruction and overexploitation [12], and is consequently listed as endangered by the IUCN [13]. Reeves’ turtles have been extirpated from much of their former range, and many of the remaining populations are associated with anthropogenically altered habitats (Bu et al., Unpublished data). Effective conservation of this species requires ecological research and specific conservation strategies based on the research findings [14].

Hibernation is a crucial aspect of the life history of the Reeves’ turtle, and successful overwintering is critical for the survival of population. Jung et al. [15] suggested that hibernation could be essential for normal follicular and egg development in Reeves’ turtles. Worryingly, Takenaka and Hasegawa [16] found many carcasses at Reeves’ turtle hibernation sites, indicating that they died during hibernation. However, there is a lack of knowledge regarding the hibernation of Reeves’ turtle in the wild, which hinders the development of effective conservation plans for this species during hibernation. Therefore, we studied wild Reeves’ turtles using radiotelemetry to determine the beginning and end dates of, and habitats selected for, hibernation, and to inform future conservation strategies for protection of this species during the hibernation season.

## 2. Materials and Methods

### 2.1. Hibernation Period Determination

This study was conducted in Qichun County (29°59′–30°40′ N, 115°12′–115°56′ E), Hubei Province, China, where we tracked 20 wild Reeves’ turtles (Table 1) from August 2021 to April 2022 using radio telemetry to determine the beginning and end of their hibernation. Tracking began in August 2021 to ensure that each individual had enough time to access their selected hibernating sites. All the turtles were weighed using electronic scales, and the straight length of their carapaces were measured using vernier calipers. A radio tracking transmitter (RI-2B, 216.000–216.999 MHz; Holohil Systems, Ltd., Carp, Ontario, Canada) was fitted to the end of the carapace of each adult wild turtle using a mixture of epoxy resin and EP hardener. The transmitter was applied between 2:00–4:00 p.m. when the temperature was high, which was conducive to the hardening of the epoxy resin and EP hardener mixture. Considering the weight and size of the turtles and the mass (8 with a mass of 6 g and 12 with a mass of 8 g), and the location of the transmitter, it did not prevent the turtles from using any microhabitat. These turtles were released into the same location where more than two individuals were caught in the wild habitat. A handheld receiver (TRX-1000S, 216.000–216.999 MHz; Wildlife Materials International, Inc., Murphysboro, IL, USA) with a three-component folding antenna (Wildlife Materials, Inc., Murphysboro, IL, USA) was used to track turtles daily. After the tracking study is completed, which is expected in October 2022, all radio tracking transmitters will be carefully removed from the carapaces of the turtles.

When a turtle stayed stationary at a site for the entire winter, this was defined as hibernation; the initial time of remaining stationary was recorded as the beginning of hibernation, and the time when the turtle left the hibernation site was recorded as the end of hibernation.

### 2.2. Habitat Selection

For each hibernating turtle, hibernation type (aquatic or terrestrial hibernation), habitat type (human-used habitats (farmed in 2021) or non-human-used habitats (not farmed in 2021)), distance from the human settlement (a settlement of more than two households), and distance from human disturbance (human activities, including grazing, agricultural activities, fish feeding, and human capture, etc.) were recorded. When turtles hibernated underground, the distance between their carapace and ground level was measured and defined as the depth of hibernation. Two different quadrat sizes (10 × 10 m and 1 × 1 m) were set at the selected sites, and the ecological factors in the quadrats were quantitatively analyzed. A random quadrat was also selected within a range of 10–50 m in a random direction from each selected quadrat. The minimum separation of 10 m ensured no overlap between the random and selected quadrats. The maximum separation of 50 m is the average home range length of Reeves’ turtle [17]. If the random site had the same habitat type as the selected site (aquatic versus aquatic, terrestrial versus terrestrial), it was recorded as the random habitat. If the habitat types differed (aquatic versus terrestrial, terrestrial versus aquatic), the random site was discarded and randomly re-positioned until the habitat types were the same. In the large terrestrial quadrats, we quantified (1) canopy (%) and (2) vegetation cover (%); in the small terrestrial quadrats, we quantified (3) canopy (%), (4) slope gradient (°), (5) herbage cover (%), (6) herbage height (cm), (7) deciduous cover (%), (8) deciduous thickness (cm), (9) distance from water (m), (10) distance from the field edge (m), (11) soil hardness (1 = easy finger insertion, 2 = difficult finger insertion, 3 = finger insertion not possible), and (12) soil moisture (1 = water could be extruded, 2 = could not be extruded into powder, 3 = could be extruded into powder). In the large aquatic quadrats, we quantified (1) water area (m^2^), (2) canopy (%), (3) herbage cover (%), (4) dissolved oxygen content, and (5) pH; in the small aquatic quadrats, we quantified (6) canopy (%), (7) herbage cover (%); (8) herbage height (cm), (9) distance from shore (m), and (10) depth of water (cm).

### 2.3. Statistical Analysis

All statistical analyses were performed using SPSS Statistics for Windows, version 18.0 (SPSS Inc., Chicago, IL, USA). The difference in the days during hibernation period between females and males was analyzed using Mann–Whitney U tests. Chi-squared tests were used to assess whether the selected and random habitats differed in habitat type, soil hardness, or soil moisture. The Kolmogorov–Smirnov test was used to test the normality of the data before the analysis. Differences between selected habitat and random habitat were analyzed using paired t-tests when data were normally distributed, while the data that were not normally distributed were analyzed using Wilcoxon signed-rank tests. When the data conformed to the normal distribution, results were expressed as the mean ± standard error (SE), and when the data did not conform to the normal distribution, they were expressed as the median (minimum, maximum). Furthermore, discriminant function analysis was performed to assess the differences in the numeric variables between the selected and random habitats and to determine the variables that best significantly separated the selected and random habitats. The significance level was set at *p* < 0.05.

## 3. Results

### 3.1. Hibernation Period

**Figure 1:**
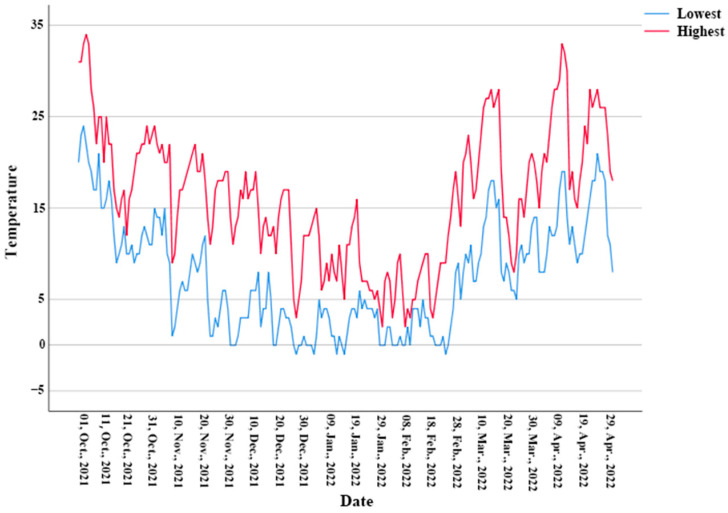
Lowest and highest temperatures during hibernation in the study area. The blue line shows the lowest temperature and the red is the highest temperature.

Of the 20 Reeves’ turtles radio telemetered in the field, 10 hibernated in aquatic habitats and 10 in terrestrial habitats. Individuals hibernating in terrestrial habitats hibernated underground, excavating and occupying burrows at an average depth of 4.27 cm (3–6 cm). The first hibernation of an individual started on 23 October (9–19 °C) and the last hibernation of an individual occurred on 14 December (4–14°C). During this time, the night temperatures were below 15 °C, although the day temperatures were still above 20 °C (Figure 1), indicating that the Reeves’ turtles will gradually enter hibernation when the lowest nightly temperature is below 15 °C. The first arousal from hibernation of an individual occurred on 6 March (7–16 °C) and the last arousal occurred on 14 April (13–19 °C). The average hibernation period was 116 days (84–155 days). During this time, daytime temperatures were above 15 °C, although nighttime temperatures were still below 10 °C (Figure 1), indicating that the Reeves’ turtles will gradually end their hibernation when the highest daily temperature is above 15 °C. In addition, there was a significant difference in hibernation days between males (105 ± 12 d) and females (124 ± 20 d) (*t* = 2.574, *df* = 17.913, *p* = 0.019). Females hibernated significantly longer than males.

### 3.2. Habitat Selection

There was a significant difference between the human-used habitats and the non-human-used habitats (*χ^2^* = 12.8, *df* = 1, *p* < 0.001), indicating that the Reeves’ turtles preferred non-human-used habitats. The distance between hibernation sites and human settlements was 150.88 ± 92.93 m (15.7–257 m), and the distance between hibernation sites and human disturbance was 53.41 ± 60.60 m (0–136 m), respectively, indicating that the Reeves’ turtles preferred hibernation sites far away from human settlements but were not far away from human disturbances.

When comparing selected versus random aquatic habitats at a broad scale (10 × 10 m) none of the variables showed a significant difference between the selected and random habitats (Table 2), whereas at a fine scale (1 × 1 m), only herbage cover was significantly different between the selected and random habitats (*t* = 2.525, *df* = 9, *p* = 0.033) (Table 2).

When comparing selected versus random terrestrial habitats at a broad scale (10 × 10 m), there was a significant difference in canopy between the selected and random quadrats (*Z* = −2.023, *p* = 0.043), but there was no significant difference in herbage cover (Table 2), whereas at a fine scale (1 × 1 m), there were significant differences in the canopy (*Z* = −2.201, *p* = 0.028), slope gradient (*Z* = −2.032, *p* = 0.042), herbage cover (*Z* = −2.379, *p* = 0.017), and distance from the field edge (*Z* = −2.524, *p* = 0.012) between the selected and random quadrats; however, there were no significant difference in other continuous variables (Table 2). In addition, there was a significant difference in soil hardness between the selected and random habitats (*χ*^2^ = 6.082, *df* = 2, *p* = 0.048), but there was no significant difference in soil moisture (*χ*^2^ = 1.726, *df* = 2, *p* = 0.422), indicating that the Reeves’ turtles preferred hibernation sites with soil of lower hardness, but had no preference in terms of moisture. Overall, this indicates that Reeves’ turtles prefer to hibernate at the soft edges of flat habitats with low canopy and high herbage cover when hibernating in terrestrial habitats, and in aquatic habitats they prefer to hibernate at sites with high herbage cover, relatively far from human settlements.

**Table 2:** Ecological factors between selected and random quadrats in hibernating Reeves’ turtles in Qichun County, Hubei Province, China.

Hibernation Type	Quadrat Size (m × m)	Factors	Mean ± SE or Median (Minimum, Maximum)		Paired T-Test or Wilcoxon Signed-Rank Test		
			Selected Habitat	Random Habitat	*t* or *Z*	*df*	*p*
Aquatic hibernation	10 × 10	Canopy (%)	0 (0, 5)	0 (0, 5)	−1.00	-	0.317
	10 × 10	Vegetation cover (%)	48.00 ± 11.23	38.00 ± 10.93	0.478	9	0.644
	10 × 10	Water area	260.10 ± 76.71	284.90 ± 76.40	−0.536	9	0.605
	10 × 10	Dissolved oxygen content	3.75 ± 0.67	4.54 ± 0.46	−1.330	9	0.216
	10 × 10	Ph	7.54 ± 0.14	7.50 ± 0.15	0.325	9	0.753
	1 × 1	Canopy (%)	0 (0, 20)	0 (0, 0)	−1.342	-	0.180
	1 × 1	Herbage cover (%)	77.00 ± 13.17	33.00 ± 14.15	2.525	9	0.033
	1 × 1	Herbage height (cm)	4.76 ± 0.84	2.45 ± 1.15	1.619	9	0.140
	1 × 1	Depth of water (cm)	74.90 ± 15.78	77.60 ± 16.82	−0.123	9	0.905
	1 × 1	Distance from shore (m)	1.90 ± 0.37	3.45 ± 0.53	−2.103	9	0.065
Terrestrial hibernation	10 × 10	Canopy (%)	0 (0, 20)	5 (0, 70)	−2.023	-	0.043
	10 × 10	Vegetation cover (%)	93.00 ± 4.23	89.00 ± 5.47	1.088	9	0.305
	1 × 1	Canopy (%)	0 (0, 30)	40 (0, 80)	−2.201	-	0.028
	1 × 1	Slope gradient (°)	0 (0, 4)	4 (0, 30)	−2.032	-	0.042
	1 × 1	Herbage cover (%)	100 (90, 100)	60 (10, 100)	−2.379	-	0.017
	1 × 1	Herbage height (cm)	22.05 ± 4.35	17.14 ± 3.21	1.121	9	0.291
	1 × 1	Deciduous cover (%)	15.00 ± 8.33	13.00 ± 5.59	0.283	9	0.783
	1 × 1	Deciduous thickness (cm)	3.23 ± 1.89	1.63 ± 0.75	0.830	9	0.428
	1 × 1	Distance from water (m)	8.79 ± 4.68	9.79 ± 5.12	−0.739	9	0.479
	1 × 1	Distance from the field edge (m)	0 (0, 1.2)	2.62 (0, 6.4)	−2.524	-	0.012

When comparing selected versus random aquatic habitats at a finer scale (1 × 1 m), the eigenvalue of stepwise discrimination was 0.952 and the canonical correlation coefficient was 0.698, which contained 100% variance. Wilks’ *λ* showed a significant difference between the selected and random habitats (Wilks’ *λ* = 0.512, *χ*^2^ = 11.368, *df* = 2, *p* = 0.003). Stepwise discriminant analysis showed that herbage coverage and depth of water could significantly distinguish the selected and random habitats, and the correct discrimination rate reached 85.0% (Table 3). When comparing selected versus random terrestrial habitats at a finer scale (1 × 1 m), the eigenvalue of stepwise discrimination was 7.560 and the canonical correlation coefficient was 0.940, which contained 100% variance. Wilks’ *λ* was significantly different between the selected and random habitats (Wilks’ *λ* = 0.117, *χ*^2^ = 35.428, *df* = 3, *p* < 0.001). Stepwise discriminant analysis showed that the herbage cover, distance from the field edge, and herbage height could significantly distinguish the selected and random habitats, and the correct discrimination rate reached 100% (Table 3).

## 4. Discussion

In this study, we observed that Reeves’ turtles enter hibernation mainly from mid-November to December, and gradually end hibernation in March or April. When the temperature rises to approximately 15 °C, it stimulates arousal in the turtles. Previous studies have shown that hibernating turtles are not comatose and remain vigilant during overwintering in cold hypoxia, allowing them to respond to light or elevated temperatures but not to vibration or increased oxygen [18,19]. More direct studies attribute arousal from terrestrial hibernation in the spring to the trigger of rising temperatures [20,21]. Males exit hibernation earlier, contrary to findings in a study on the desert tortoise (*Gopherus agassizii*) [22], probably because Reeves’ turtles start mating immediately after hibernation, and males must forage to obtain nutrients to cope with widespread migration in search of mates [23].

Reeves’ turtles overwinter in a variety of habitats, ranging from aquatic to terrestrial. For aquatic hibernation, selected sites had high herbage cover (77%), including fish ponds, marshes, and abandoned ponds (ponds not being used for farming). Aquatic overwintering allows turtles to avoid exposure to fluctuating air temperatures [24] while maintaining metabolic depression over an extended period. However, overwintering in an aquatic environment imposes significant limitations on organisms because atmospheric oxygen is limited or unavailable during ice cover periods, which can last for five months or more [25,26]. Reeves’ turtles hibernated in ponds and marshes that had low levels of dissolved oxygen, indicating that they were anoxia-tolerant. Wintering under ice in such conditions, or in oxygen-deprived mud, requires adaptation to prolonged periods of hypoxia or anoxia [4]. There are many similar anoxia-tolerant species, such as painted turtles (*Chrysemys picta*) [6,27] and snapping turtles (*Chelydra serpentina*) [28], which can survive for over 100 days in oxygen-deprived water at 3 °C. In addition, aquatic hibernacula sites covered with herbage are important to Reeves’ turtles, protecting them from predators and freezing.

Half of the Reeves’ turtles’ selected terrestrial hibernation sites had more herbage cover and were close to the field edge. In contrast to aquatic hibernation, the higher predation risk associated with terrestrial hibernation may pose a greater risk than the physical challenge of leaving the water to seek refuge on land, if the refuge site is not at risk of freezing, flooding, or dehydration [4,10,29]. Reeves’ turtles excavate and occupy burrows, which are important for thermoregulation [30,31] and protect individuals from human activities and predators [32]. The hibernation site of one turtle was burned; however, the individual was unharmed. In addition, cattle are frequently herded in these areas in the autumn, which damages the soil, thus decreasing its suitability for the turtles [30]. However, the edges of fields are often overlooked by cattle; therefore, Reeves’ turtles hibernate near the edge, occupying better conditions. Reeves’ turtles hibernate in low-canopy open fields, which is possibly unfavorable for their survival. Walden [33] suggested that open habitats without canopy cover have harder soil and greater temperature variation, which are more variable than forest habitats. Moreover, it may increase the risk for turtles from late autumn, winter, and early spring farming activities [34]. However, in our study area, wild boars were abundant in forest areas and rarely appeared in fields; therefore, hibernating in fields may have reduced their risk of predation by wild boars. In addition, there were no farming activities in these areas during the hibernation period. Therefore, hibernation under high herbage cover reduces predation risk and prevents freezing.

In the current study, no individuals died during hibernation, confirming that turtles rarely die during hibernation [4]. However, Takenaka and Hasegawa [16] found that many Reeves’ turtles died during hibernation, which may be due to physical effects, such as pond drying, prolonged ice penetration below a hibernaculum, or protracted anoxia [4].

In conclusion, Reeves’ turtles mainly hibernate in abandoned ponds or lands. The terrestrial hibernation sites had more herbage cover and were close to the field edge, and the aquatic hibernation sites were covered with herbage, which provides shelter and protection and thermal stability for the turtles during hibernation. Based on the importance of these habitats for the species, we suggest vigilantly protecting this unique resource to provide Reeves’ turtles with secure hibernaculum sites and avoiding redevelopment of these areas during hibernation.

## Figures and Tables

**Table 1:** Hibernating Reeves’ turtle tracked during field surveys in Qichun County, Hubei Province, China.

Number	Weight (g)	Carapace Straight Length (mm)	Sex
1	97.8	87.4	♂
2	325.4	133.4	♀
3	380.9	141.56	♀
4	430.9	145.86	♀
5	886.1	170.76	♀
6	612.6	160.64	♀
7	311.6	131.78	♀
8	350.4	129.68	♀
9	115.2	87.4	♂
10	149.5	103.7	♂
11	166.3	101.68	♂
12	110.1	90.38	♂
13	135.6	100.4	♂
14	637.2	156.48	♀
15	440	134.3	♀
16	406.9	136.08	♀
17	118.3	87.52	♂
18	352.9	138.5	♀
19	871.6	178.74	♀
20	147.5	100.18	♂

**Table 3:** Stepwise discriminant analysis between selected and random habitats in hibernating Reeves’ turtles in Qichun County, Hubei Province, China.

Hibernation Type	Factors	Discriminant Coefficients	Wilks’ *λ*	*F*	*p*
Aquatic hibernation	Herbage cover	0.047	0.756	5.814	0.027
	Depth of water	0.028	0.512	8.089	0.003
Terrestrial hibernation	Herbage cover	0.106	0.533	15.759	0.001
	Distance from the field edge	1.175	0.145	50.155	<0.001
	Herbage height	−0.042	0.117	40.323	<0.001

## Data Availability

The data presented in this study are available in the Supplementary Material.

## Supplementary Materials

The following supporting information can be downloaded at: https://www.mdpi.com/article/10.3390/ani12182411/s1, Table S1. Habitat Selection. Table S2. Lowest and highest temperatures.
